# Supplementary material for: Drug repurposing for aging research using model organisms
Source: Aging Cell. 2017 Jun 16;16(5):1006–15. doi: 10.1111/acel.12626 (PMC5595691; doi:10.1111/acel.12626)
Supplement: Supplementary file 7 — Data S1 Zip‐Archive of all report cards. [file ACEL-16-1006-s007.zip › RC_REA.pdf]

REA

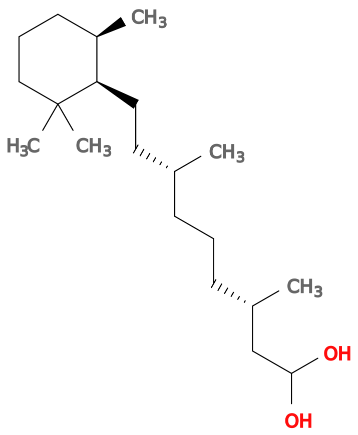

Database identifiers

ChEMBLCompound	CHEMBL38
CHEBI	15367
eMolecules	1934590

Ranking

	Rank	Score
Drosophila	365/697	0.412
C. elegans	NA	NA

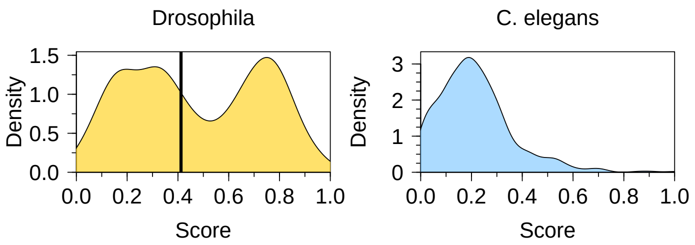

	Ageing implication	Domain conservation	Binding site conservation	Binding affinity	Bioavailability	Lipinski	Promiscuity	Purchasability	Drug approval	**Total**
Drosophila	0.624	0.649	0.771	0.936	(0.9)	-0.05	-0.001	0.1	0.1	0.412
C. elegans	NA	NA	NA	NA	NA	NA	NA	NA	NA	NA

Names

- **all-trans-retinoic acid**
- Acide retinoique (French) (DSL)
- Agn 100335
- Atralin
- Avita
- Renova
- Ro 1-5488
- Tretin M
- tretinoin
- Tretinoine (French) (EINECS)
- Vesanoid
- Vitamin A acid

Roles

antineoplastic agent, keratolytic drug, antioxidant, signalling molecule, retinoid X receptor agonist, anti-inflammatory agent, AP-1 antagonist, retinoic acid receptor agonist, human metabolite, Retinoic acid receptor agonist

Status

Approved drug (according to ChEMBL)	Yes
Number of Rule of 5 violations	1
Binding affinity to original target in log units (RF-Score prediction)	7.69
Burns *C. elegans* bioavailability prediction	-2.74

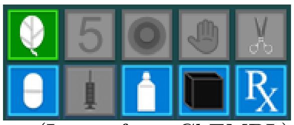

(Image from ChEMBL)

Compound Target Characteristics

Retinoic acid receptor RXR-alpha

Best gene implication in ageing for this target family came from gene Q0VJ96 via mapping the annotation from RGD 3610 annotated in RGD 2014-03-11. Annotation GO 7568 (aging) was Inferred from Expression Pattern

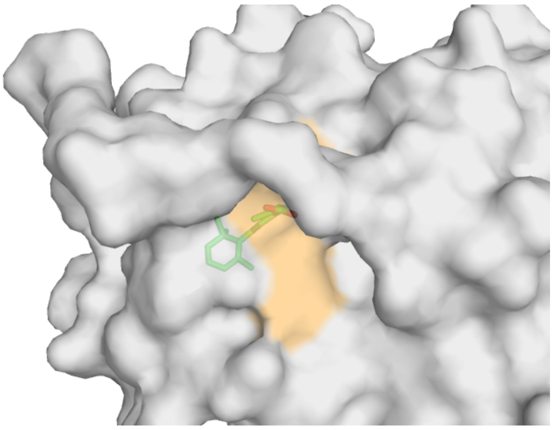

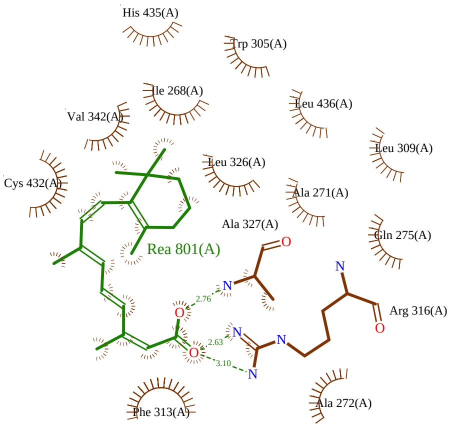

protein	amino acids contacts (binding site)
PDB:1fby:chainA:P19793	I A A Q W N L F R L A V I C H L
PDB:1xls:chainA:P19793	I A A Q W N L F R L A V I C H L
PDB:2acl:chainA:P19793	I A A Q W N L F R L A V I C H L
PDB:3fal:chainA:P19793	I A A Q W N L F R L A V I C - -
PDB:3fc6:chainA:P19793	I A A Q W N L F R L A V I C H L
PDB:3oap:chainA:P19793	I A A Q W N L F R L A V I C H L
PDB:3uvv:chainB:P19793	I A A Q W N L F R L A V I C H L
PDB:4nqa:chainA:P19793	I A A Q W N L F R L A V I C H L
tr:B3KY83:B3KY83_HUMAN	I A A Q W N L F R L A V I C H L
sp:P19793:RXRA_HUMAN	I A A Q W N L F R L A V I C H L
tr:F1D8Q5:F1D8Q5_HUMAN	I A A Q W N L F R L A V I C H L
tr:Q6P3U7:Q6P3U7_HUMAN	I A A Q W N L F R L A V I C H L
tr:Q0VJ96:Q0VJ96_RAT	I A A Q W N L F R L A V I C H L
tr:Q6LC96:Q6LC96_MOUSE	I A A Q W N L F R L A V I C H L
sp:P28700:RXRA_MOUSE	I A A Q W N L F R L A V I C H L
tr:A2AJP1:A2AJP1_MOUSE	I A A Q W N L F R L A V I C H L
sp:P20153:USP_DROME	L V V Q W I L F R L N V I C H L

protein	whole protein ident	simil	domain-based ident	simil	contact-based ident	simil
PDB:1fby:chainA:P19793	1.0	1.0	1.0	1.0	1.0	1.0
PDB:1xls:chainA:P19793	1.0	1.0	1.0	1.0	1.0	1.0
PDB:2acl:chainA:P19793	1.0	1.0	1.0	1.0	1.0	1.0
PDB:3fal:chainA:P19793	1.0	1.0	1.0	1.0	0.88	0.75
PDB:3fc6:chainA:P19793	1.0	1.0	1.0	1.0	1.0	1.0
PDB:3oap:chainA:P19793	1.0	1.0	1.0	1.0	1.0	1.0
PDB:3uvv:chainB:P19793	1.0	1.0	1.0	1.0	1.0	1.0
PDB:4nqa:chainA:P19793	1.0	1.0	1.0	1.0	1.0	1.0
tr:B3KY83:B3KY83_HUMAN	0.79	0.79	1.0	1.0	1.0	1.0
sp:P19793:RXRA_HUMAN	1.0	1.0	1.0	1.0	1.0	1.0
tr:F1D8Q5:F1D8Q5_HUMAN	1.0	1.0	1.0	1.0	1.0	1.0
tr:Q6P3U7:Q6P3U7_HUMAN	0.9	0.9	1.0	1.0	1.0	1.0
tr:Q0VJ96:Q0VJ96_RAT	0.97	0.99	1.0	1.0	1.0	1.0
tr:Q6LC96:Q6LC96_MOUSE	0.92	0.93	1.0	1.0	1.0	1.0
sp:P28700:RXRA_MOUSE	0.97	0.99	1.0	1.0	1.0	1.0
tr:A2AJP1:A2AJP1_MOUSE	0.97	0.99	1.0	1.0	1.0	1.0
sp:P20153:USP_DROME	0.37	0.61	0.4	0.66	0.69	0.77

usp (FBgn0003964) associated phenotypes

circadian rhythm defective, germline clone, heat sensitive, hypoactive, lethal - all die before end of pupal stage, lethal - all die during P-stage, lethal - all die during first instar larval stage, maternal effect, neuroanatomy defective, partially, rescuable maternal effect, somatic clone, some die during pupal stage, touch sensitivity defective

(Information from FlyBase)

usp (UniProt:P20153) annotation

Function: Receptor for ecdysone. May be an important modulator of insect metamorphosis. Plays an important part in embryonic and post-embryonic development. Binds to ecdysone response elements (ECRES) such as in the promoter region of s15 chorion gene.
Subunit: Heterodimer of USP and ECR. Only the heterodimer is capable of high-affinity binding to ecdysone. (PubMed:8247157).
Subcellular location: Nucleus.
Domain: Composed of three domains: a modulating N-terminal domain, a DNA-binding domain and a C-terminal ligand-binding domain.

(Information from UniProt)

Retinoic acid receptor RXR-alpha

Best gene implication in ageing for this target family came from gene Q0VJ96 via mapping the annotation from RGD 3610 annotated in RGD 2014-03-11. Annotation GO 7568 (aging) was Inferred from Expression Pattern

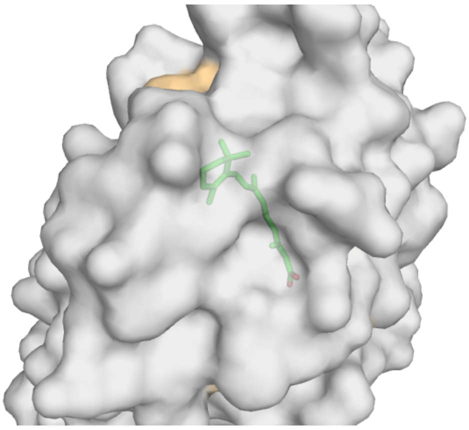

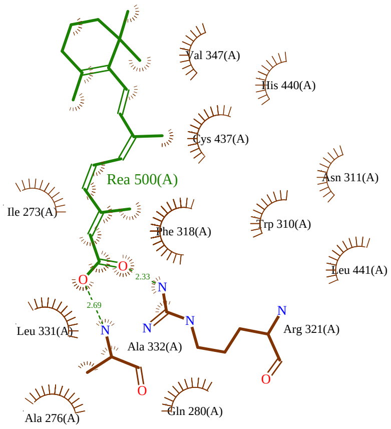

protein	amino acids contacts (binding site)
PDB:1xdk:chainA:P28700	I A Q W N F R L A V C H L
tr:B3KY83:B3KY83_HUMAN	I A Q W N F R L A V C H L
sp:P19793:RXRA_HUMAN	I A Q W N F R L A V C H L
tr:F1D8Q5:F1D8Q5_HUMAN	I A Q W N F R L A V C H L
tr:Q6P3U7:Q6P3U7_HUMAN	I A Q W N F R L A V C H L
tr:Q0VJ96:Q0VJ96_RAT	I A Q W N F R L A V C H L
tr:Q6LC96:Q6LC96_MOUSE	I A Q W N F R L A V C H L
sp:P28700:RXRA_MOUSE	I A Q W N F R L A V C H L
tr:A2AJP1:A2AJP1_MOUSE	I A Q W N F R L A V C H L
sp:P20153:USP_DROME	L V Q W I F R L N V C H L

	whole protein		domain-based		contact-based	
protein	ident	simil	ident	simil	ident	simil
PDB:1xdk:chainA:P28700	1.0	1.0	1.0	1.0	1.0	1.0
tr:B3KY83:B3KY83_HUMAN	0.78	0.78	1.0	1.0	1.0	1.0
sp:P19793:RXRA_HUMAN	0.97	0.99	1.0	1.0	1.0	1.0
tr:F1D8Q5:F1D8Q5_HUMAN	0.97	0.99	1.0	1.0	1.0	1.0
tr:Q6P3U7:Q6P3U7_HUMAN	0.87	0.88	1.0	1.0	1.0	1.0
tr:Q0VJ96:Q0VJ96_RAT	0.99	1.0	1.0	1.0	1.0	1.0
tr:Q6LC96:Q6LC96_MOUSE	0.94	0.94	1.0	1.0	1.0	1.0
sp:P28700:RXRA_MOUSE	1.0	1.0	1.0	1.0	1.0	1.0
tr:A2AJP1:A2AJP1_MOUSE	1.0	1.0	1.0	1.0	1.0	1.0
sp:P20153:USP_DROME	0.37	0.65	0.4	0.66	0.69	0.74

usp (FBgn0003964) associated phenotypes

circadian rhythm defective, germline clone, heat sensitive, hypoactive, lethal - all die before end of pupal stage, lethal - all die during P-stage, lethal - all die during first instar larval stage, maternal effect, neuroanatomy defective, partially, rescuable maternal effect, somatic clone, some die during pupal stage, touch sensitivity defective

(Information from FlyBase)

usp (UniProt:P20153) annotation

Function: Receptor for ecdysone. May be an important modulator of insect metamorphosis. Plays an important part in embryonic and post-embryonic development. Binds to ecdysone response elements (ECRES) such as in the promoter region of s15 chorion gene.
Subunit: Heterodimer of USP and ECR. Only the heterodimer is capable of high-affinity binding to ecdysone. (PubMed:8247157).
Subcellular location: Nucleus.
Domain: Composed of three domains: a modulating N-terminal domain, a DNA-binding domain and a C-terminal ligand-binding domain.

(Information from UniProt)
